# 

**Published:** 1820-12-01

**Authors:** 


### Contents.

Vol. 1. No. 3. DECEMBER, 1820.

Art. I.

MUCOUS MEMBRANE OF THE LUNGS.

1. Dr. Hastings on Diseases of the Mucous Membrane of the Lungs
2. M. Broussais sur les Phlegmasies Chroniques
3. Mr. Alcock on the Mucous Membranes
4. Dr. Harrison on Eruptive Fevers - - - 341

II.

Dr. Prichard on Epidemic Fever - - - 381

III.

Medical Transactions of the College of Physicians, Vol. VI. 387

Art. 1. Dr. Yeats's Case of Strangulated Hernia - - - 389

2. Dr. Baillie on Paraplegia - - - 391

3. Dr. Kerckhoff's Medical Observations - - - 394

4. Dr. Cholmeley's Case of Malformation - - - 395

5. Dr. Carter on Warm Climates - - - 396

6. Dr. Latham on Solanum Tuberosum - - - 400

7. Dr. Powell on Painful Affections of the Bowels - - 402

8. Mr. Webb on the Plague of Egypt - - - 407

IV.

CHOLERA MORBUS.

Art. 1. Calcutta Medical Board on Cholera
2. Mr. Corbyn's Letter to Sir Gilbert Blane - - - 409

V.

Mr. Pring on the General Indications which relate to the Laws of Organic Life - - - 416

VI.

Mr. Wilson's Lectures on the Structure and Physiology of the Parts composing the Skeleton - - - 427

VII.

Dr. George Gregory's Elements of the Theory and Practice of Physic - - - 435

VIII.

Dr. Hutchinson's Dissertation on Infanticide - - - 444

IX.

SUPPLEMENTAL REVIEW of PRACTICAL MEDICINE, digested and arranged - - - - - - - - - - - - - - - - - - - - - - - - 452

Art. 1. Mr. Earle on the Meatus Auditorius, p. 452.—2. Dr. Dean on the Ear, 445.—3. Mr. Alcock on Congenital Division of the Palate, with a plate, 456.—4. Gogiran's remarkable disease of the Heart and Liver, 458.—5. Dr. Gardiner's Case of Cardi-hepatic Disease, 461.—6. Baron Larrey on Empyema succeeding Wounds of the Chest, 462.—7. M. Vaidy on the Employment of Caustic Ammonia in Thoracic Affections, 472.—8. Dr. Hennen and Mr. Henderson's Cases of Hydrothorax, 474.—Dr. Romero's Cases of Puncturing the Pericardium, 477.—9. Dr. Bailey's Case of Fungoid Tumour in the Abdomen, 479.—10. Dr. McSweeny's Case of Spicula of Bone in the Stomach, 480.—11. Mr. Chevalier on Relaxed Rectum, 481.—12. Mr. Bampfield's Case of Entero-Epiplocele, 484.—13. Dr. Granville's Case of Ovario-Gestation, 485.—14. Dr. Campbell on Uterine Hæmorrhage, 486.—15. Mr. Mathias's Case of Hernia with Hæmorrhage, 488.—16. Baron Larrey on Wounds of the Intestines, 489.— 17. Mr. Bell on Bursting of the Urethra, 493.—18. Dr. Dewees on Dysmenorrhœa, 495.—19. Dr. Colles on Fracture of the Neck of the Femur, 498.—20. Baron Larrey on Luxation of the Thigh, 500.—21. Dr. Fletcher on Tabes Mesenterica, 500.—22. Mr. Wardrop on Inordinate Irritability, 501.—23. Inflammation of Arteries, 502.—24. Substitutes for Cinchona, 505.—25. Dr. Morison on Cutaneous Diseases, 506.—26. Case of Obliterated Aorta, 507.—27. Dr. Burrows on Melancholia, 508.—28. Poisoning by Oxymurias Hydrargyri, 509.—29. Unguentum Hydrargyri in Erysipelas, 510.—30. On Infusum Digitalis, 511.

X.

Retrospective Review of Medical and Surgical Science in the United States of America, during the last 30 Years - - 512

XI.

Dr. MERRIMAN's Synopsis of the various kinds of Difficult Parturition, with Practical Remarks on the Management of Labours - - - - - - - - - - - - - - - - - - - - - - - - 526

XII.

Mr. BINGHAM's Practical Essays on Strictures of the Urethra, Diseases of the Testicles, &c. &c. - - - - - - - - - - - 544

XIII.

Dr. PARIS's Pharmacologia, 4th Edition enlarged - - - - - 550

XIV.

Dr. JOHNSON's Reply to certain Reviewers - - - - - - - - - 551

MISCELLANIES, LIST OF BOOKS FOR REVIEW, &c. - - - - - 557

